# Influence of Enzymatically Hydrophobized Hemp Protein on Morphology and Mechanical Properties of Bio-Based Polyurethane and Epoxy Foams

**DOI:** 10.3390/polym15173608

**Published:** 2023-08-31

**Authors:** Guillem Ferreres, Sílvia Pérez-Rafael, Angela Gala Morena, Tzanko Tzanov, Liudmyla Gryshchuk

**Affiliations:** 1 Grup de Biotecnologia Molecular i Industrial, Universitat Politècnica de Catalunya, Edifici Gaia, TR14, Rambla Sant Nebridi, 22, 08222 Terrassa, Spain; guillem.ferreres@upc.edu (G.F.); silvia.perez.rafael@upc.edu (S.P.-R.); angela.gala.morena@upc.edu (A.G.M.); tzanko.tzanov@upc.edu (T.T.); 2 Leibniz-Institut für Verbundwerkstoffe GmbH, Erwin-Schrödinger-Straße 58, 67663 Kaiserslautern, Germany

**Keywords:** polyurethane and epoxy composite foams, hemp protein, laccase-assisted hydrophobization, bio-fillers, mechanical properties

## Abstract

Biomass fillers offer the possibility to modify the mechanical properties of foams, increasing their cost-effectiveness and reducing their carbon footprint. In this study, bio-based PU (soft, open cells for the automotive sector) and epoxy (EP, hard, closed cells for construction applications) composite foams were prepared by adding pristine and laccase-mediated lauryl gallate-hydrophobized hemp protein particles as filler (HP and HHP, respectively). The fillers were able to modify the density, the mechanical properties and the morphology of the PU and EP foams. The addition of HP filler increases the density of PU foams up to 100% and significantly increases the σ values by 40% and Emod values. On the other hand, the inclusion of the HHP as filler in PU foams mostly results in reduced density, by almost 30%, and reduced σ values in comparison with reference and HP-filled foams. Independently from filler concentration and type, the biomass increased the Emod values for all foams relative to the reference. In the case of the EP foams, the tests were only conducted for the foams filled with HHP due to the poor compatibility of HP with the EP matrix. HHP decreased the density, compressive strength and Emod values of the composites. For both foams, the fillers increased the size of the cells, while reducing the amount of open cells of PU foams and the amount of closed cells for EP foams. Finally, both types of foams filled with HHP reduced the moisture uptake by 80 and 45%, respectively, indicating the successful hydrophobization of the composites.

## 1. Introduction

Polyurethane foams (PUFs) are extensively utilized across various industries due to their versatility, lightweight nature, and exceptional thermal insulation properties. Despite the domination of polyurethane foams on the foam market [1], the development of other foam types, for example, polystyrene (PS), poly(vinyl chloride) (PVC), polyethylene (PE), polypropylene (PP) or poly(methyl methacrylate) [1], phenolic [2,3,4], and (bio)-epoxy foams [5,6,7,8,9], is spreading continuously. Although each type of foam presents its own advantages, density, thermal stability, and mechanical properties are key parameters for its ultimate application. One possible way to improve the density as well as mechanical properties of PU and epoxy foams is the introduction of filler(s) [10,11]. Moreover, filler can reduce costs while maintaining acceptable performance levels, especially if the filler components are generally cheaper than the base materials [12,13,14,15].

A promising filler material that has gained attention for use in PUFs is hemp biomass. Hemp is a fast-growing plant that can be cultivated without excessive water or chemical supplementation, making it cost-effective relative to other crops [16]. It is considered a renewable resource, aligning with sustainability goals and reducing reliance on non-renewable materials [17]. Additionally, the strong and stiff fibers or particles derived from hemp can enhance the foam’s tensile strength, flexural strength, and impact resistance, thereby increasing the foam’s overall performance [18,19].

However, hemp and other types of biomass fillers do present some challenges. Hemp moisture adsorption capacity can negatively affect the long-term dimensional stability and mechanical properties of foams (including polyurethane), as well as the reaction kinetics and the foam expansion during the production of the material [20,21,22,23]. Moreover, biomass fillers may face issues related to degradation, durability, and dispersion within the polyurethane matrix [24,25,26]. They can also contribute to increased flammability and hinder the flame retardant properties of the foam, requiring additional additives or treatments to comply with fire safety regulations [27].

To address these concerns, hydrophobized hemp biomass fillers offer several improvements. The incorporation of hydrophobic fillers has the potential to enhance flame retardant effectiveness, improve compatibility with the matrix, promote better dispersion, and result in greater consistency of performance. Moreover, the application of this modified biomass could contribute to increasing the mechanical properties of the foam [28,29,30].

Herein, we modified hemp protein by laccase-catalyzed oxidative grafting of lauryl gallate (LG)—a phenolic compound with an alkaline chain. Grafting was carried out by adapting a previously reported method to produce hydrophobized cellulose and wool [31,32,33]. In this process, biomass was pre-activated enzymatically using acetosyringone. This mediator facilitates the laccase-assisted oxidation of chemical groups that would otherwise be inaccessible to the enzyme. By implementing this approach, we were able to achieve the LG grafting onto the hemp biomass in a waterborne reaction without the need for hazardous reagents used in other chemical hydrophobization reactions, such as periodate, or harsh conditions, such as combustion methods. The hydrophobized biomass was used as filler for polyurethane and epoxy foams, and its influence on the foam properties was investigated.

## 2. Materials and Methods

### 2.1. Materials and Reagents

Polycarbonatediols Cardyon^®^ LC 05 (made using Covestro’s CO_2_-technology integrating up to 20 percent CO_2_ into polyol, OH n = 53.5 KOH/g) and ETERNACOLL UT-200 (OH n = 56 KOH/g) were supplied by Covestro (Leverkusen, Germany) and UBE Corporation Europe (Castellón de la Plana, Spain), respectively. Poly(propylene glycol) 4000, Polyethylene glycol 600, Aspartic acid, Formic acid, Dibutyltin dilaurate, Tween 80, Poly(methylhydrosiloxane), lauryl gallate (LG) and carboxymethylcellulose sodium salt were purchased from Sigma-Aldrich (St. Louis, MO, USA). Exolit^®^ OP 560 (co-reactive flame retardant with an OH value of 450 mg KOH/g) was supplied by Clariant (Muttenz, BL, Switzerland). Ortegol 500 was supplied by Evonik (Essen, Germany). LED-103 (reactive, acid blocked catalyst, OH n = 2405 KOH/g) and Niax silicone L-6164 were supplied by Momentive Performance Materials Inc. (Antwerp, Belgium) Iso 133/6 poly(4,4′-Diphenylmethandiisocyanat) with 32% of NCO groups and Ongronat CO5700—PMDI/PPG-prepolymer with 8.5% NCO content were supplied by BASF (Ludwigshafen am Rhein, LU, Germany) and BorsodChem (Kazincbarcika, Hungary), respectively.

Bio-based epoxy resin SR Greenpoxy 56 and amino-hardener SZ 8525 (from Sicomin Epoxy systems) were purchased from Time Out Composite oHG, Bornheim-Sechtem, Germany. Epoxidized Cardanol Cardolite^®^ NC-513 and CNSL Novolac resin NX-4001 were supplied by Cardolite Corporation. Novozymes (Bagsværd, Denmark) supplied fungal laccase Novozym 51003 from *Myceliophthora thermophile* (EC1.10.3.2). 3′,5′-dimethoxy-4′-hydroxyacetophenone (acetosyringone) was obtained from ACROS Organics (Geel, Belgium). Hemp protein residues obtained from the oil-pressing process of hempseeds were kindly provided by Kroppenstedter Olmühle (Kroppenstedt, Germany). All reagents for foam preparation were used without any additional purification.

### 2.2. Hydrophobization of Hemp Protein

The hydrophobization of hemp protein powder was accomplished by an enzymatic, laccase-mediated functionalization with LG in a bioreactor Labfors 5 (Infors HT, Bottmingen, Switzerland), following a previously described protocol, with some modifications [23]. First, 7.5 mM of acetosyringone was dissolved in 2 L of 50 mM sodium acetate buffer at pH 5.5. Then, 10 mg/mL of biomass was added to the mixture. Upon complete dissolution of the reagents, the hemp protein was pre-activated using laccase (13 U/mL) for 1 h at 50 °C. Subsequently, LG solution containing 40 vol.% ethanol was introduced into the mixture to initiate the grafting process on the biomass to a final concentration of 6 mM of LG and 20 vol.% of ethanol. After 2 h of reaction, the modified hemp powder was separated by centrifugation at 10,000× *g* for 30 min in order to eliminate the unreacted compounds. The resulting pellet was frozen at −80 °C and subsequently freeze-dried to obtain the final functionalized hemp protein product.

### 2.3. Polyurethane Foam Preparation

Bio-based PU foams were prepared using a one-step method. At first, a solution consisting of a blend of polyols Cardyon^®^ LC 05, Eternacoll UT-200, Poly(propylene glycol) 4000, and Polyethylene glycol 600 was prepared. To the obtained solution, aspartic and formic acids were added as blocking agents; LED-103 and Dibutyltin dilaurate were added as blowing and gelling catalysts, respectively. Niax silicone L-6164 and Ortegol 500 were added as cell-openers, and Tween 80 used as a bio-based co-surfactant. A water solution of 2.5 wt% carboxymethylcellulose was used as chemical blowing agent and bio-based co-surfactant. Exolit^®^ OP 560 and Poly(methylhydrosiloxane) were added as co-reactive flame retardant and blowing agent, respectively. The resulting mixture (denoted as Component A) was mixed for 20 min at 2000 rpm for homogenization.

In the case of preparation of filled foam, the filler was added in appropriate concentration to Component A before homogenization. More specifically, the obtained fillers were added at the following concentrations: 0.25; 0.5; 1, 1.5; 2, 2.5 and 3 wt%. For foams with filler amount ≥3.5 wt%, post-reaction shrinking of more than 4% was observed. That is why these foams were not considered for further testing. Moreover, in general, for HHP-filled foams, we observed lower shrinking in comparison with HP-filled foams.

Next, an appropriate amount ([NCO]/[OH] = 1.05) of Component B, a blend of Iso 133/6 and Ongronat CO5700, was added to Component A, and their combination was stirred with mechanical stirring for 20 s at 2000 rpm. Immediately afterwards, the resultant mixture was transferred into an open cylindrical mold, allowing free rising at room temperature. For the sake of brevity, the produced composite foams were named as PU_Name_y, where “Name” is the abbreviation of corresponding filler and “y” refers to wt% of the filler added to the PU matrix. For example, the “HP” in the sample name PU_HP_1 referred to hemp protein as filler, and the “1” indicated the wt% of HP added in the PU matrix, while PU_HHP_y was used for foams with hydrophobized hemp protein, respectively. Unfilled PU foam named PU_Ref was used as reference. Table 1 reports the amounts of reagents used in PU formulation for 100 g of total foam.

### 2.4. Epoxy Foam Preparation

Bio-based epoxy foams were prepared using a one-step method. At first, an appropriate amount of SR GreenPoxy 56 as base resin, CNSL Novolac resin NX-4001 as co-resin and Cardolite^®^ NC-513 as co-reactive diluent were thoroughly mixed together at 50–55 °C for 10 min with a propeller mixer at 2000 rpm. After that, the appropriate amount of hardener SZ 8525 was added to the resin blend and mixed at 2000 rpm for 2 min. In the last step, Poly(methylhydrosiloxane) as blowing agent was added, and all components were mixed for 1 min at 2000 rpm. Afterwards, the resultant reactive mixture was immediately transferred into an open Al mold for free-rise, kept at RT for 1 h, and then post-cured at 70 °C for 4 h.

In the case of filled foam preparation, the appropriate amount of filler (0.25; 0.5; 1, 1.5; 2, 2.5 and 3 wt%) was added in resin blend before homogenization. The produced composite foams were named as Epoxy_Name_y, where “Name” is the abbreviation of the corresponding filler, and “y” refers to the wt% of filler added to the epoxy matrix. For example, in a sample named Epoxy_HHP_1, “HHP” refers to the filler, and “1” indicates the wt% of HHP added in the epoxy matrix. Unfilled epoxy foam named as Epoxy_Ref was used as reference. For composite epoxy foams, only HHP was used as filler because of the significantly worse dispersibility of HP in resin. Table 2 reports the amounts of reagents used in epoxy formulations for 100 g of total foam.

### 2.5. Characterization of the Hydrophobized Hemp Protein

To evaluate the enzymatic grafting modification, FTIR analysis of the biomass was recorded over the 4000−650 cm^−1^ range, performing 64 scans with a PerkinElmer Spectrum 100 (PerkinElmer, Waltham, MA, USA). The baseline was corrected, and the spectra were normalized using the PerkinElmer Spectrum software v.1.0, with the maximum absorbance intensity value serving as the reference. The hydrophobicity of the biomass was determined using the sessile drop method. A layer of hemp powder was applied to a glass support, and then a 2 µL water droplet was casted onto the biomass. Subsequently, the contact angle of the drop was measured using a Drop Shape Analyzer (Krüss, Hamburg, Germany).

### 2.6. Polyurethane and Epoxy Foam Characterization

#### 2.6.1. Characterization of Density and Mechanical Properties

The density of PU and epoxy foams was determined at 23 °C with 50% relative humidity [34]. The density value reported is the average value of 10 specimens with size 30 mm × 30 mm × 30 mm (length × width × thickness).

Mechanical compressive strength of PU/epoxy foams was determined according to [35] and carried out through a Zwick 1445 Retroline machine (ZwickRoell GmbH and Co. KG, Berlin, Germany). The following parameters were used for measurement: initial load 0.5 N, E-modulus velocity 10 mm/min, testing velocity 10%/min, maximal deformation 70%. Compressive strength at 10% and 40% strain and according to values of compressive modulus were performed. Then, 10 specimens were tested, and an average value was taken along with the standard deviation.

#### 2.6.2. Determination of Moisture Uptake

Hydrophobicity of filled PU and epoxy foams was determined by moisture uptake test in humidity camera at 23 °C and relative humidity of 90%. Foam samples before testing were dried at 40 °C to constant weight and then placed in the humidity camera. At intervals of 24 h, the samples were weighed to control weight increases due to water absorption. The experiment was considered fully completed if the last three weight measurements showed a weight with a maximal difference of 0.00001 g.

## 3. Results and Discussion

### 3.1. Characterization of the Hydrophobized Hemp Protein

**Figure 1:**
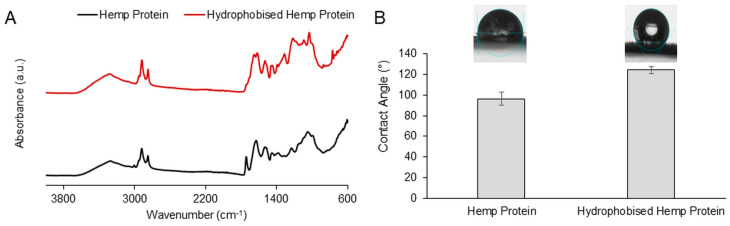
FTIR spectra of hemp protein (black) and hydrophobized hemp protein (red) (**A**) and contact angle of the biomass before and after the hydrophobization (**B**).

The laccase-assisted method to graft lauryl gallate onto hemp protein yielded hydrophobized biomass. This modification was evaluated through FTIR and contact angle. The spectrum of the hydrophobized biomass showed an increase in the signals at ~2919 and ~2857 cm^−1^ compared to the unmodified sample, corresponding to the C-H stretching absorption of the lauryl gallate. Furthermore, the signal associated with the C=C-C of the aromatic ring at ~1619 cm^−1^ also increased in the modified hemp spectrum. Additionally, the LG moieties caused the appearance of signals in the regions of ~1200 and ~700 cm^−1^ due to C-O and C-H bonds, respectively (Figure 1A) [33]. To assess the hydrophobicity of the modified material, the contact angle was measured and compared with the pristine biomass. The contact angle of the hemp after treatment increased from 96.4° to 124.2° (Figure 1B), thereby confirming the successful hydrophobization of the material. LG is an ester of gallic acid and dodecanol; the reaction with laccase couples the phenolic groups with the ones present in the hemp protein, exposing the long hydrocarbon chain. The non-polar nature of these chains repels the water, conferring to the biomass hydrophobic properties.

**Figure 2:**
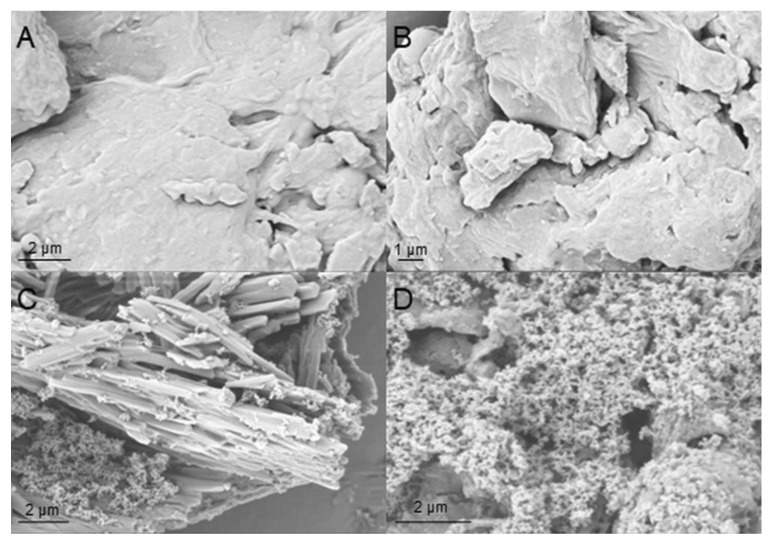
SEM images of pristine (**A**,**B**) and hydrophobized (**C**,**D**) hemp protein under 20,000× and 30,000× magnification, respectively.

In order to test the influence of hydrophobic modification on the morphology of hemp protein particles, the biomass was analyzed by SEM. As is visible from the SEM images, the hydrophobization had a significant influence on the surface structure of the hemp protein particles (Figure 2). The HP SEM images displayed a smooth surface, while the HHP presented attached on the surface nano-structures due to the modification with LG. Similar surface morphology changes have been previously described in lignocellulosic biomass modified with this compound [36].

### 3.2. Polyurethane Foam Characterization

**Figure 3:**
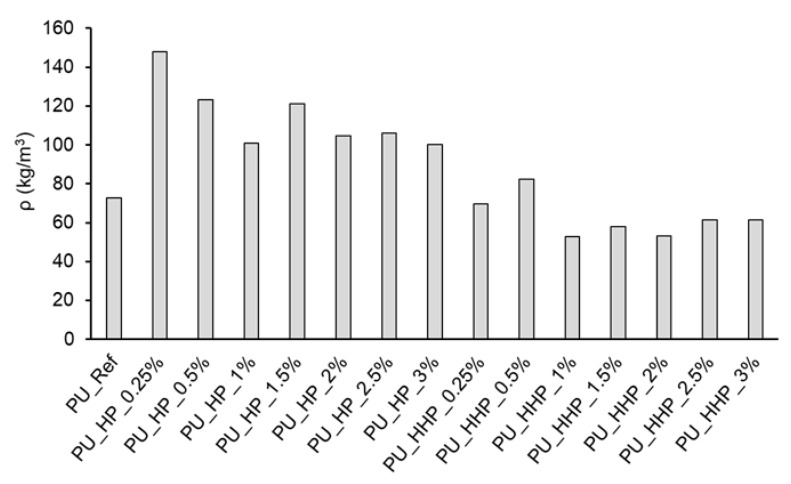
Density of bio-based polyurethane foams filled with pristine (HP) and hydrophobized hemp protein (HHP) (max. standard deviation 4.16%).

In order to test the influence of hemp protein hydrophobization on the polyurethane foams’ mechanical properties, determination of the filled foam density and the assessment of compressive strength values were carried out. Before foam preparation, fillers were dispersed in Component A (detailed description in Section 2.3). For foams filled with hemp protein, the density increased relative to the reference foam. Modified hemp protein-filled PU foams presented lower density than the reference foams and the HP-filled ones (Figure 3). It must be noted that hydrophobized filler demonstrated significantly better dispersibility in polyol blend.

**Figure 4:**
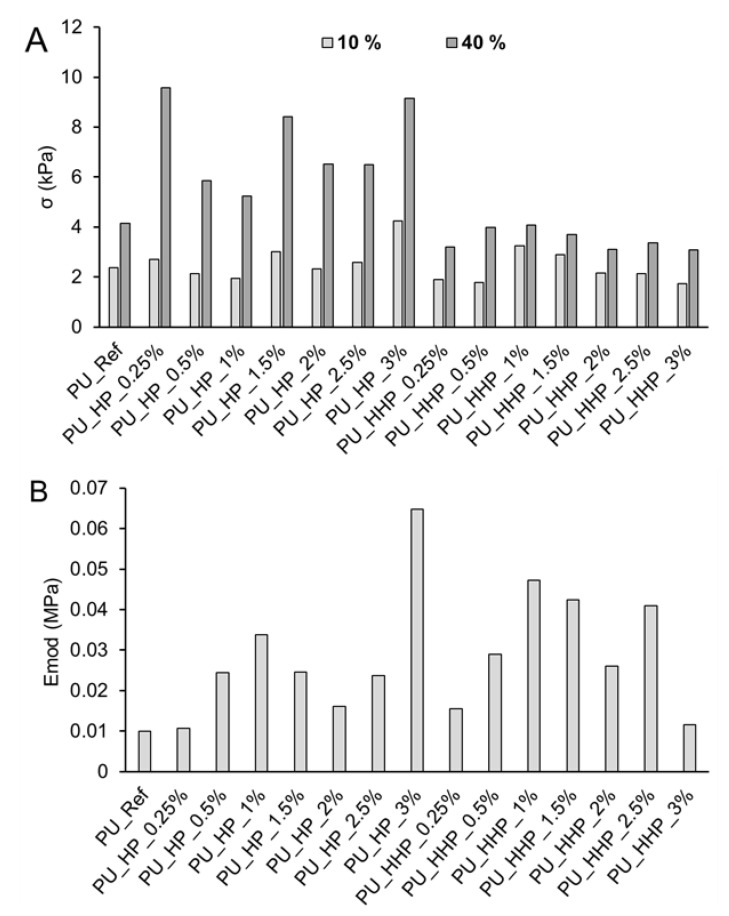
Compressive strength values at 10% and 40% deformation (**A**) and Emod values (**B**) for reference polyurethane foam and foam filled with pristine and hydrophobized hemp protein (max. standard deviation 6.85% and 9.01% for σ and Emod values, respectively).

Testing of mechanical properties demonstrated a significant increase in σ40% values for HP-filled PU foams; however, the σ10% values did not differ significantly. Only the foams with 3 wt% of filler displayed an increase in both σ10% and σ40% values in comparison with the reference in ca. 100%. PU foams filled with hydrophobized hemp protein exhibited the highest σ10% and σ40% values at 1 wt% filler content, surpassing the values of the reference foams. In general, addition of HHP as filler in PU foams mostly resulted in decreased density and compressive strength in comparison with reference and the HP-filled foams (Figure 4A). Values of Emod increased for foams with both filler types independent of the filler concentration. HP-filled foams with 3 wt% of filler presented the highest value (increasing 6.5-fold in comparison with the reference). The foams filled with HHP, with the exception of the foam with a filler content of 3 wt%, presented higher Emod values than those of the HP-filled ones, which indicates better resistance to deformation by external forces (Figure 4B).

**Figure 5:**
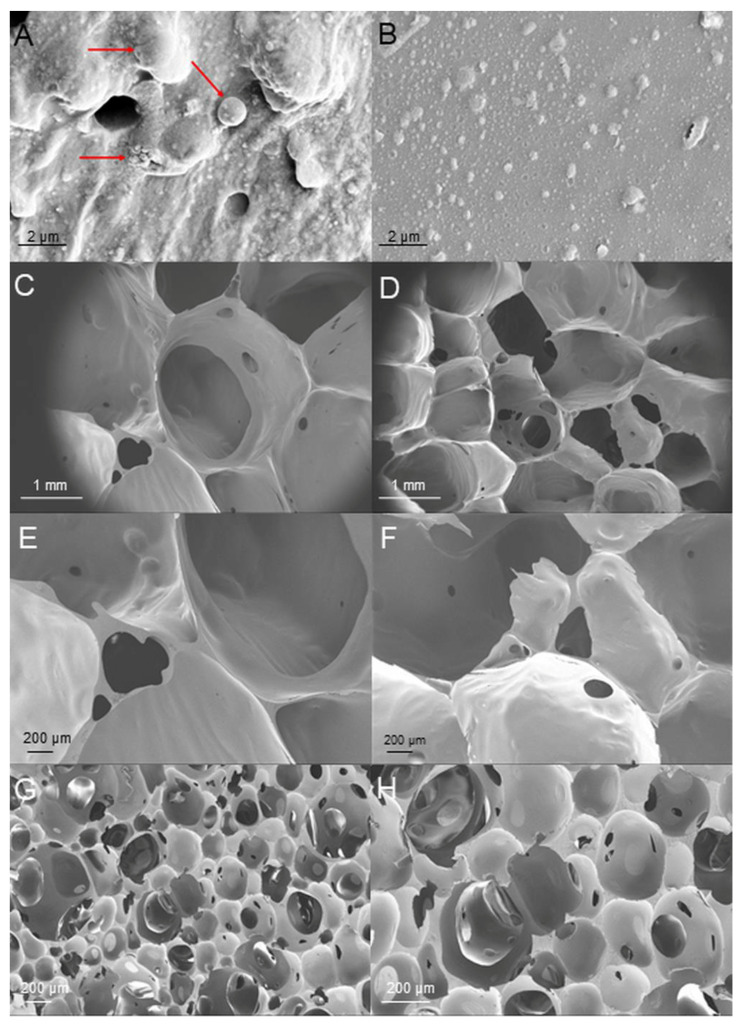
SEM images of polyurethane foams filled with 3 wt% of pristine (**A**,**C**,**E**) and 3 wt% of hydrophobized (**B**,**D**,**F**) hemp protein under 50×, 100× and 20,000× magnification, respectively. (**G**,**H**) SEM images of the reference polyurethane foam (100× and 200× magnification).

Such significant differences in the mechanical properties of foams filled with hydrophobized and non-hydrophobized hemp protein can be explained by the different morphology of the foams. The higher hydrophilicity of the pristine fillers reduced their dispersibility in the polyurethane matrix, producing bigger agglomerates. Moreover, at higher concentrations of the pristine HP (for example, 3 wt%), the agglomerates were embedded in the matrix (Figure 5A, areas marked with red arrows). For the foam filled with the same concentration of HHP, there was an obviously better dispersion of the filler in the polyurethane matrix, presenting agglomerates of much smaller size (Figure 5B). At the same time, bigger cells were formed in the HP-filled foams (Figure 5C,E) relative to the HHP-filled ones (Figure 5D,F). A similar trend in open-cell flexible PU foams—an increase in cell size with increasing hydrophilicity of the filler—was reported by Sung et al. [30]. This effect can be explained by improved compatibility of the hydrophobic or, as in our case, hydrophobized biomass with the matrix, which leads to an increase in interfacial adhesion. Furthermore, the addition of both fillers in the PU foams increased the foams’ cell size in comparison with the non-filled foams and decreased the amount of open cells (Figure 5G,H). Because the morphology of the foams has a direct influence on the mechanical properties, analyzing the data from the mechanical tests and SEM studies, it can be concluded that the introduction of fillers increases the cell size and σ and Emod values.

However, the presence of agglomerates may cause a reduction in these properties at higher reinforcement content. A similar tendency has been reported for soft PU composite foams filled with SiO_2_ [37].

### 3.3. Epoxy Foam Characterization

**Figure 6:**
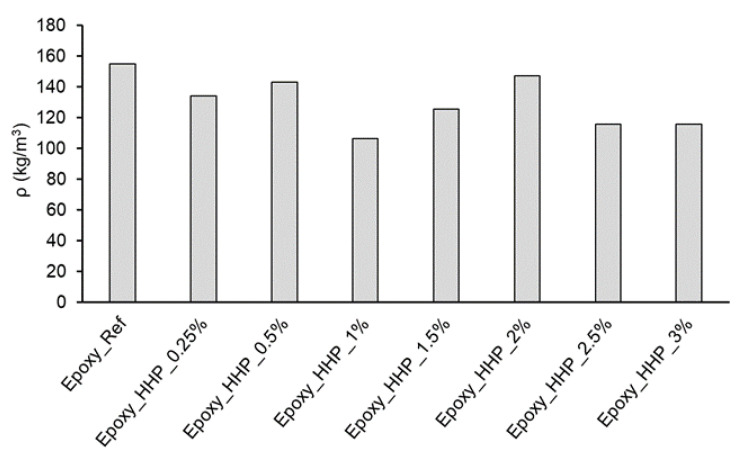
Density of reference epoxy foam and foam filled with hydrophobized hemp protein (max. standard deviation 4.98%).

In the case of epoxy foams, only HHP fillers were used in the formulation due to the poor dispersibility of HP in the resin. The addition of the HHP fillers into the epoxy mixture (hard foams with closed cells) reduced the density of the foams in comparison with the reference. The tendency is similar to that of polyurethane foams filled with HHP, as the foam filled with 1 wt% HHP was the one with the lowest density (Figure 6).

**Figure 7:**
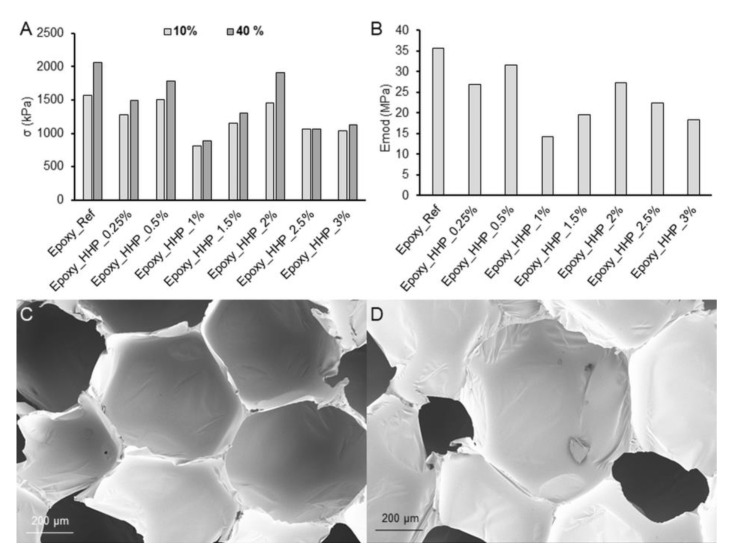
Compressive strength values at 10% and 40% deformation (**A**) and Emod values (**B**) for reference and epoxy foam filled with hydrophobized hemp protein (max. standard deviation 6.85% and 7.54% for σ and Emod values, respectively); SEM images of the reference (**C**) and filled (1 wt% of HHP) epoxy foam (**D**) under 200× magnification.

The compressive strength test showed that the inclusion of HHP in epoxy foam decreased both σ10% and σ40% values with increasing filler amount (Figure 7A). The same tendency was observed for Emod values (Figure 7B). The epoxy foam filled with 1 wt% of HHP presented the lowest density, the lowest values of compressive strength at 10% and 40% deformation as well as Emod.

The SEM analysis demonstrated that the fillers increased the size of the cells and decreased the number of closed cells (Figure 7C,D). This tendency has been previously described; the density and mechanical properties of rigid polyurethane foams filled with precipitated silica were decreased with increased filler loading due to cell damage [38].

### 3.4. Moisture Uptake of the Foams

Finally, the moisture uptake of both types of foams was measured. The addition of pristine hemp protein to polyurethane foam increased the hydrophobicity insignificantly. At the maximal tested amount (3 wt%) of the HP-filler, moisture uptake decreased by 18% in comparison with reference. At the same time, the addition of the hydrophobized hemp protein resulted in decreasing the moisture uptake by 80% in comparison with the reference. The same tendency was observed for the epoxy foams filled with the HHP. Although the non-filled epoxy foam was strongly hydrophobic, increasing the hydrophobized filler amount resulted in decreasing the moisture uptake by 45%, showing the feasibility of the hydrophobization of hemp protein (Table 3).

## 4. Conclusions

Hydrophobized hemp protein was successfully obtained through a laccase-mediated modification process. To the best of our knowledge, HHP was used for the first time as a filler for PU and EP foams. The introduction of HHP into the formulation of either PU or EP foams resulted in decreasing the foams’ density, which is an advantage for the application of such materials in lightweight construction and thermal insulation areas. For the PU foams, the inclusion of HPP in their formulation increased the Emod, potentially reducing the deformation of the composite. In the case of EP foams, HPP reduced the compressive strength and the Emod. Incorporation of the HPP as filler led to a decrease in the number of open cells in PU foams. As a result, an increase in the Emod of PU foams was observed. For EP foams, the introduction of HHP filler reduced the number of closed cells, which led to a decrease in Emod; from another point of view, such foams could be suitable for better sound absorption. Furthermore, both foams presented reduced moisture uptake when filled with modified biomass, demonstrating the effective hydrophobization of the composites. Finally, differences in the influence of the hydrophobized hemp protein on the morphology and, as result, mechanical properties of open- and closed-cell foams pose new questions and challenges. Such different tendencies with increasing (for PU foams) and decreasing (for EP foams) amounts of closed cells and increasing cell size with the addition of filler, as well as significant changes in density and mechanical properties, especially Emod values, requires further research, which is already planned by the authors: (i) synthesis and investigation of nanoparticles of HHP; (ii) investigation of dispersing methods for selection of the method most suitable for agglomerate-free nano-composite foam preparation; (iii) investigation of the influence of bio-based nano-fillers on the density and mechanical properties of composite foams.

## Figures and Tables

**Table 1:** Amounts of reagents used in PU formulations for 100 g of total foam.

Component	Amount, g
Cardyon^®^ LC 05	16.37
Poly(propylene glycol)4000	4.01
Eternacoll UT-200	3.04
Polyethylene glycol 600	1.96
Water + CMC_2.5%	2.50
Aspartic acid	1.02
Exolit^®^ OP 560	5.73
Dibutyltin dilaurate	1.60
Formic acid	1.39
LED-103	0.05
Tween 80	1.02
Niax silicone L-6164	1.02
Ortegol 500	1.23
Poly(methylhydrosiloxane)	0.82
iso 133/6	19.24
Ongronat CO5700	39.00
	100.00

**Table 2:** Amounts of reagents used in EP formulations for 100 g of total foam.

Component	Amount, g
SR GreenPoxy 56	72.46
Cardolite^®^ NX-4001	3.62
Cardolite^®^ NC-513	3.62
SZ 8525	18.12
Poly(methylhydrosiloxane)	2.18
	100.00

**Table 3:** Moisture uptake for reference and filled PU and epoxy foams.

Sample	Moisture Uptake,%	Sample	Moisture Uptake,%	Sample	Moisture Uptake,%
PU_Ref	5.06 ± 0.21			Epoxy_Ref	1.23 ± 0.27
PU_HP_0.25%	4.99 ± 0.19	PU_HHP_0.25%	4.04 ± 0.50	Epoxy_HHP_0.25%	1.10 ± 0.58
PU_HP_0.5%	4.97 ± 0.19	PU_HHP_0.5%	3.20 ± 0.44	Epoxy_HHP_0.5%	0.95 ± 0.19
PU_HP_1%	4.91 ± 0.21	PU_HHP_1%	2.81 ± 0.34	Epoxy_HHP_1%	0.90 ± 0.42
PU_HP_1.5%	4.90 ± 0.14	PU_HHP_1.5%	2.12 ± 0.31	Epoxy_HHP_1.5%	0.85 ± 0.30
PU_HP_2%	4.84 ± 0.23	PU_HHP_2%	1.86 ± 0.60	Epoxy_HHP_2%	0.82 ± 0.50
PU_HP_2.5%	4.23 ± 0.16	PU_HHP_2.5%	1.28 ± 0.38	Epoxy_HHP_2.5%	0.79 ± 0.23
PU_HP_3%	4.18 ± 0.30	PU_HHP_3%	1.04 ± 0.29	Epoxy_HHP_3%	0.68 ± 0.14

## Data Availability

The data presented in this study are available on request from the corresponding author.
